# Neuregulin-1 attenuates cognitive function impairments in a transgenic mouse model of Alzheimer's disease

**DOI:** 10.1038/cddis.2016.30

**Published:** 2016-02-25

**Authors:** J Ryu, B-H Hong, Y-J Kim, E-J Yang, M Choi, H Kim, S Ahn, T-K Baik, R-S Woo, H-S Kim

**Affiliations:** 1 Department of Pharmacology and Biomedical Sciences, College of Medicine, Seoul National University, 103 Daehakro, Jongno-gu, Seoul, Republic of Korea; 2 Department of Anatomy and Neuroscience, College of Medicine, Eulji University, Daejeon, Republic of Korea; 3 Seoul National University College of Medicine, Bundang Hospital, Bundang-Gu, Sungnam, Republic of Korea; 4 Neuroscience Research Institute, College of Medicine, Seoul National University, 103 Daehakro, Jongno-gu, Seoul, Republic of Korea

## Abstract

The neuregulin (NRG) family of epidermal growth factor-related proteins is composed of a wide variety of soluble and membrane-bound proteins that exert their effects via the tyrosine kinase receptors ErbB2-ErbB4. In the nervous system, the functions of NRG1 are essential for peripheral myelination, the establishment and maintenance of neuromuscular and sensorimotor systems and the plasticity of cortical neuronal circuits. In the present study, we report that an intracerebroventricular infusion of NRG1 attenuated cognitive impairments in 13-month-old Tg2576 mice, an animal model of Alzheimer's disease (AD). In addition, according to Golgi-Cox staining, NRG1 rescued the reduction in the number of dendritic spines detected in the brains of Tg2576 mice compared with vehicle (PBS)-infused mice. This result was also corroborated *in vitro* as NRG1 attenuated the oligomeric amyloid beta peptide_1-42_ (A*β*_1-42_)-induced decrease in dendritic spine density in rat primary hippocampal neuron cultures. NRG1 also alleviated the decrease in neural differentiation induced by oligomeric A*β*_1-42_ in mouse fetal neural stem cells. Collectively, these results suggest that NRG1 has a therapeutic potential for AD by alleviating the reductions in dendritic spine density and neurogenesis found in AD brains.

Neuregulin 1 (NRG1) is a member of growth and differentiation factors that contain the epidermal growth factor-like domain. NRG1 has been originally linked with schizophrenia^1^ and has been implicated in neurodevelopmental processes including neuronal differentiation and synapse formation.^2^ Studies regarding NRG treatment on cells show that NRG1 can promote neurite outgrowth in hippocampal and thalamic primary neurons.^3, 4^ A more recent study by Krivosheya and colleagues^5^ demonstrated that soluble NRG1 treatment on hippocampal primary neurons facilitates synapse maturation and dendritic arborization.

NRG1 is highly expressed in the developing brain and has the above-mentioned roles, and remains to be widely expressed in the adult nervous system.^6^ In addition, NRG1 mRNA is highly expressed in the CA3 area of the hippocampus.^7^ Heterozygous NRG1-KO mice exhibit impairments in prepulse inhibition and working memory.^8^ A recent study revealed that NRG1 signaling regulates fear memory by maintaining high GABAergic activity in amygdala.^9^ NRG1 has important roles in the maintenance of brain circuits and is involved in functions such as synaptic plasticity and modulation of neurotransmitter release. In adult hippocampal slices, soluble NRG1 suppresses the induction of long-term potentiation (LTP) at Schaffer collateral-CA1 synapses,^10^ while neutralization of endogenous NRG1 with ecto-ErbB4 enhances hippocampal LTP.^11^ Biochemical and electrophysiological evidence indicate that NRG1 stimulates GABA release in response to depolarization in the prefrontal cortex.^6^

The activity of NRG1 is highly dependent on the cleavage performed by *β*-site of amyloid precursor protein-cleaving enzyme (BACE1).^12^ BACE1 also participates in the cleavage of amyloid precursor protein (APP), leading to the production of amyloid beta peptide (A*β*) which is critical for the pathogenesis of Alzheimer's disease (AD).^13^ BACE1-KO mice have been reported to share distinctly equivalent features to heterozygous NRG1-KO mice, such as prepulse inhibition impairments, novelty-induced hyperactivity, supersensitivity to MK-801 and cognitive deficits.^14^ AD is neuropathologically characterized by the presence of neuritic plaques principally composed of A*β* and neurofibrillary tangles.^15^ The pathogenesis of AD could be explained by a loss in neural plasticity^16^ that may adversely affect dendritic arborizations, synaptic remodeling, LTP, axonal sprouting, synaptogenesis and neurogenesis.

On the basis of the relevance between NRG1 and AD, we found that soluble NRG1 can prevent A*β*_1-42_-induced impairment of LTP in a previous study.^17^ In addition, NRG1 has been shown to alleviate the cytotoxic effects of A*β*_1-42_, Swedish APP and C-terminal fragments of APP,^18, 19^ proposing that NRG1 may have protective effects in AD. In order to further investigate the significance of NRG1 in AD, we investigated the effects of NRG1 in both *in vitro* and *in vivo* experiments in this study.

## Results

### NRG1 attenuates the impairments in learning and memory in 13-month-old Tg2576 mice

**Figure 1:**
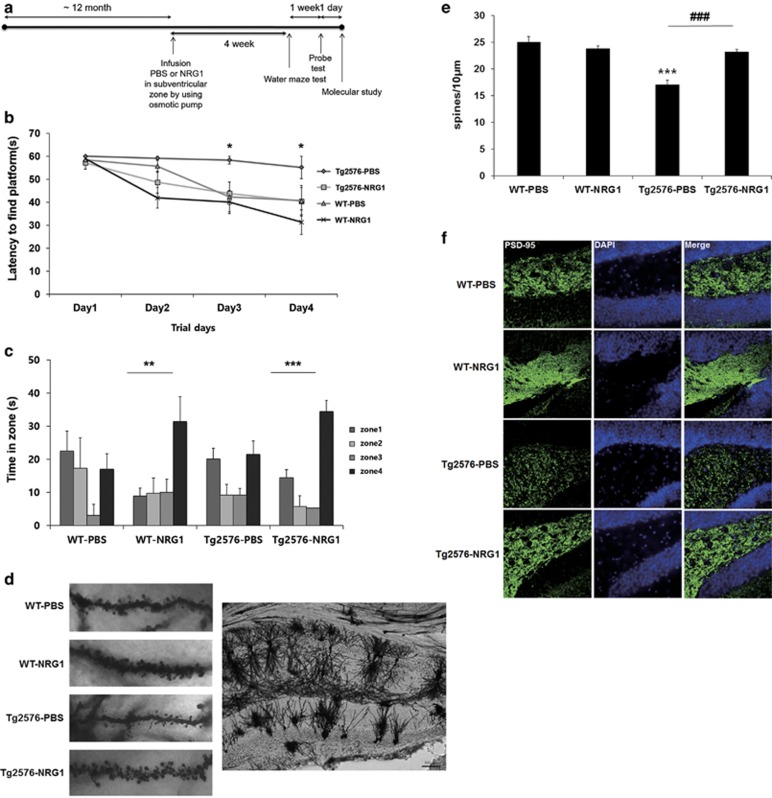
Intraventricular infusion of NRG1 peptide attenuates learning and memory deficits in Tg2576 mice. (**a**) An experimental schematic of intraventricular infusion of NRG1 peptide using an osmotic pump is shown. PBS or NRG1 was infused into 12-month-old Tg2576 mice and their WT littermates. The Morris water maze test was performed 4 weeks after the osmotic pump insertion surgery. (**b**) Animals were required to find a submerged platform (12 cm in diameter, 35 cm in height) in the pool using spatial cues. Three training trials per day were conducted for four consecutive days, in which the initial placement of the mice into the maze was changed for trial and for each group. The latency to escape to the hidden platform was recorded for each training session. Significant differences were detected between the Tg2576-PBS group and the Tg2576-NRG1 group on day 3 and day 4 of the Morris water maze task. (*n*=5 for WT-PBS, 6 for WT-NRG1, 3 for Tg2576-PBS and 4 for Tg2576-NRG1) per group; *P<0.05 based on a one-way ANOVA, *post hoc* analysis Fisher's LSD. (**c**) Forty-eight hours after the final trial session, a single probe trial was conducted. The escape platform was removed, and each mouse was allowed to swim for 60 s in the maze. NRG1-infused WT or Tg2576 mice remained significantly longer in zone 4 than the remaining zones (zones 1, 2 and 3) (one-way ANOVA, **P<0.01, **P<0.001). (**d**) Golgi-Cox staining was performed on the mouse brains using a FD Rapid GolgiStain Kit (FD Neuro Technologies) according to the manufacturer's instructions. Right; a representative image of CA1 subregion of hippocampus from WT-PBS-infused mice. Left; representative images of dendritic spines are provided for the WT-PBS, WT-NRG1, Tg2576-PBS and the Tg2576-NRG1-infused mice. (**e**) For each group, two to three brains from each group were subjected to Golgi-Cox staining (WT-PBS: *n*=3, WT-NRG1 *n*=2, Tg2576-PBS *n*=2, Tg2576-NRG1 *n*=2). Pyramidal neurons with countable morphology in the CA1 region were selected from the slides (WT-PBS: *n*=23, WT-NRG1 *n*=14, Tg2576-PBS *n*=11, Tg2576-NRG1 *n*=16), and two to four dendrites were counted to score each neuron). Tg2576 mice exhibited a reduced dendritic spine density compared with WT mice (WT-PBS: 25.03±1.04 spines/10 *μ*m, *n*= 23; Tg2576-PBS: 17.06±7.09 spines/10 *μ*m, *n*=11, ***P<0.001). NRG1 infusion in Tg2576 mice alleviated the decrease in dendritic spine density in the CA1 region (23.24±0.44, *n*=16, ***P<0.001 compared with Tg2576-PBS). (**f**) Representative images displaying the immunoreactivity against PSD95 for the four groups. Representative images were selected in a blinded manner

First, we tested whether NRG1 improved the impairments in learning and memory observed in Tg 2576 mice, an animal model of AD. We infused phosphate-buffered saline (PBS) or NRG1 into the lateral ventricle of 12- month-old wild-type (WT) or Tg2576 mice via an osmotic pump that was implanted using a stereotaxic apparatus. A diagram summarizing the experimental procedure is provided in Figure 1a.

Four weeks after the infusion, we determined the spatial learning and memory capabilities using the Morris water maze test in the WT-PBS (*n*=5), WT-NRG1 (*n*=6), Tg2576-PBS (*n*=3)- and Tg2576-NRG1 (*n*=4)-infused mice. The group of NRG1-infused 13-month-old Tg2576 mice exhibited shorter latencies (day 3: 43.83±4.98, day 4: 40.41±5.92, *P<0.05) than the group of PBS-infused Tg2576 mice (day 3: 58.33±1.67, day 4: 55.11±4.89) of the same age on days 3 and 4 of the learning sessions (Figure 1b). On day 6, a probe test was performed in which the platform was removed, and we evaluated the average latency in zone 4, which is the zone where the platform had been placed during training. NRG1-infused WT and Tg2576 mice remained in zone 4 significantly longer than other zones (zones 1, 2 and 3) (**P<0.01, ***P<0.001, respectively, Figure 1c). However, no significant differences were detected for the remaining two groups of mice with respect to the times spent in each zone (Figure 1c).

To determine whether the NRG1-mediated cognitive improvements were due to changes of the A*β* levels, we used western blotting to assay the levels of A*β* in hippocampus of WT and Tg2576 mice infused with the vehicle (PBS) or NRG1. No significant differences in A*β* levels were detected between the groups (Supplementary Figure 1), indicating that A*β* levels were not affected by NRG1.

### NRG1 rescues the decrease in dendritic spine density in Tg2576 mice based on Golgi-Cox staining

Synaptic failure is one of the pathological processes involved in AD.^20^ We evaluated the dendritic spines via Golgi-Cox staining in WT-PBS, WT-NRG1, Tg2576-PBS- and Tg2576-NRG1-infused mice. For each group, two to three brains from each group were subjected to Golgi-Cox staining (WT-PBS: *n*=3, WT-NRG1 *n*=2, Tg2576-PBS *n*=2, Tg2576-NRG1 *n*=2). Pyramidal neurons with countable morphology in the CA1 region were selected from the slides (WT-PBS: *n*=23, WT-NRG1 *n*=14, Tg2576-PBS *n*=11, Tg2576-NRG1 *n*=16), and two to four dendrites were counted to score each neuron. As shown in Figures 1d and e, Tg2576 mice exhibited a reduced dendritic spine density compared with WT mice (WT-PBS: 25.03±1.04 spines/10 *μ*m, *n*= 23; Tg2576-PBS: 17.06±7.09 spines/10 *μ*m, *n*= 11, ***P<0.001). NRG1 infusion in Tg2576 mice alleviated the decrease in dendritic spine density in the CA1 region (23.24±0.44, *n*=16, ***P<0.001 compared with Tg2576-PBS) (lower left of Figure 1d).

### NRG1 alleviates the decrease in PSD95 immunoreactivity in Tg2576 mice

Postsynaptic density protein 95 (PSD95) is an important factor that contributes to synaptic formation, and it has been proposed to be a molecular scaffold for receptors and the cytoskeleton in synapses.^21, 22, 23^ Furthermore, it has been reported that PSD95 immunoreactivity was reduced in AD patients and Tg2576 mice.^21, 24, 25^

We compared the immunoreactivity against PSD95 via immunohistochemistry in the WT-PBS, WT-NRG1, Tg2576-PBS- and Tg2576-NRG1-infused mice. NRG infusion alleviated the decrease in PSD95 expression in the hippocampus in Tg2576 mice (Figure 1f).

### NRG1 rescues the decrease in dendritic spine density induced by oligomeric A*β*_1-42_ in rat primary hippocampal neuron cultures

**Figure 2:**
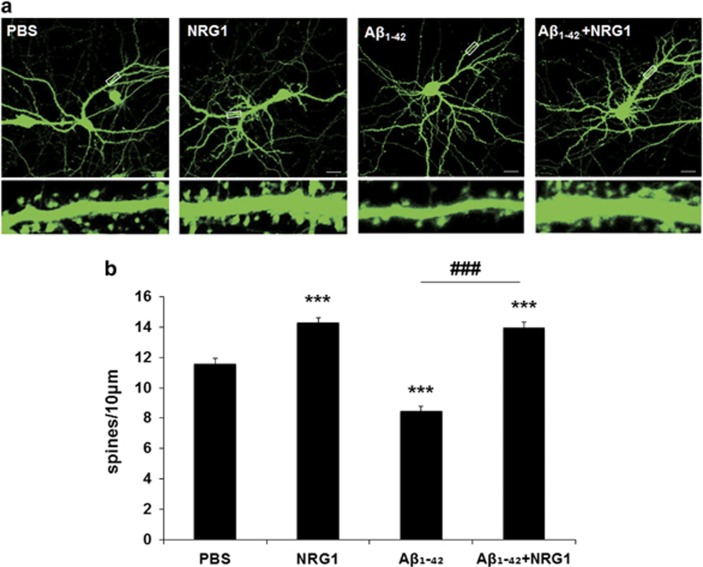
NRG1 rescues the decrease in dendritic spine density induced by oligomeric A*β*_1-42_ in rat primary hippocampal neurons. (**a**) Representative images of dendritic spines in primary hippocampal neurons at DIV 17 after treatment with oligomeric A*β*_1-42_, (at DIV 13) either alone or with PBS or NRG1 (at DIV 14) are shown. The outlined dendritic segment (upper) is enlarged to depict the spine morphology (bottom). Scale bars represent 20 *μ*m (upper) and 5 *μ*m (lower). (**b**) Quantification of spine density (40–80 *μ*m of secondary dendritic spines from the soma) at DIV 17 after transfection with mGFP in primary hippocampal neurons at DIV 12. Treatment with 250 nM A*β*_1-42_ significantly decreased dendritic spine numbers, the number per 10 *μ*m of dendrites (8.445±0.347, *n*=16, ***P<0.001), compared with PBS-treated controls (11.548±0.3875, *n*=18). Co-treatment with A*β*_1-42_ plus 10 nM NRG1 alleviated the decrease in dendritic spines induced by A*β*_1-42_ (13.960±0.367, *n*=19, ^###^*P*<0.001) compared with the A*β*_1-42_ treatment (8.445±0.347, *n*=16). Statistical analysis was performed via one-way ANOVA followed by Fisher's LSD *post hoc* test; the data are expressed as the mean±S.E.M.

The majority of the excitatory synapses in the mammalian CNS are formed on dendritic spines, and spine morphology and distribution are critical for synaptic transmission, synaptic integration and plasticity.^26, 27^ To examine the effects of NRG1 at the synapse, primary hippocampal neurons were transfected with IRES-mGFP vector at days per *in vitro* (DIV) 12 and treated with 10 nM NRG1 at DIV 14. We then determined the number of dendritic spines at DIV 17 (Figure 2a). Treatment with 10 nM NRG1 for 3 days significantly upregulated dendritic spine numbers by 23.6% (14.272±0.347/10 *μ*m, *n*=19, ***P<0.001) compared with the PBS-treated neurons (11.548±0.387/10 *μ*m, *n*=18, Figure 2a). These results are consistent with previous studies demonstrating the effects of NRG1 on dendritic spines.^28, 29, 30^

The presence of soluble A*β* oligomers in the brain is highly correlated with synaptic dysfunction in AD.^20^ Oligomeric A*β*_1-42_ treatment has been reported to decrease dendritic spine density.^31^ We confirmed that treatment of rat primary hippocampal neurons with 250 nM oligomeric A*β*_1-42_ for 4 days induced a significant decrease in dendritic spine density (8.445±0.347/10 *μ*m, *n*=16, ***P<0.001), compared with the PBS-treated neurons (11.548±0.387/10 *μ*m, *n*=18). Treatment with 10 nM NRG1 1 day after treatment of oligomeric A*β*_1-42_ for 3 days (from DIV 14 to DIV 17) restored the decrease in dendritic spine density induced by the treatment with oligomeric A*β*_1-42_ (13.960±0.367/10 *μ*m, *n*=19, ^###^*P*<0.001), compared with the oligomeric A*β*_1-42_ plus PBS-treated neurons (8.445±0.347/10 *μ*m, *n*=16) (Figures 2a and b).

### NRG1 has an attenuating effect against oligomeric A*β*_1-42_-induced reduction of neurite outgrowth

**Figure 3:**
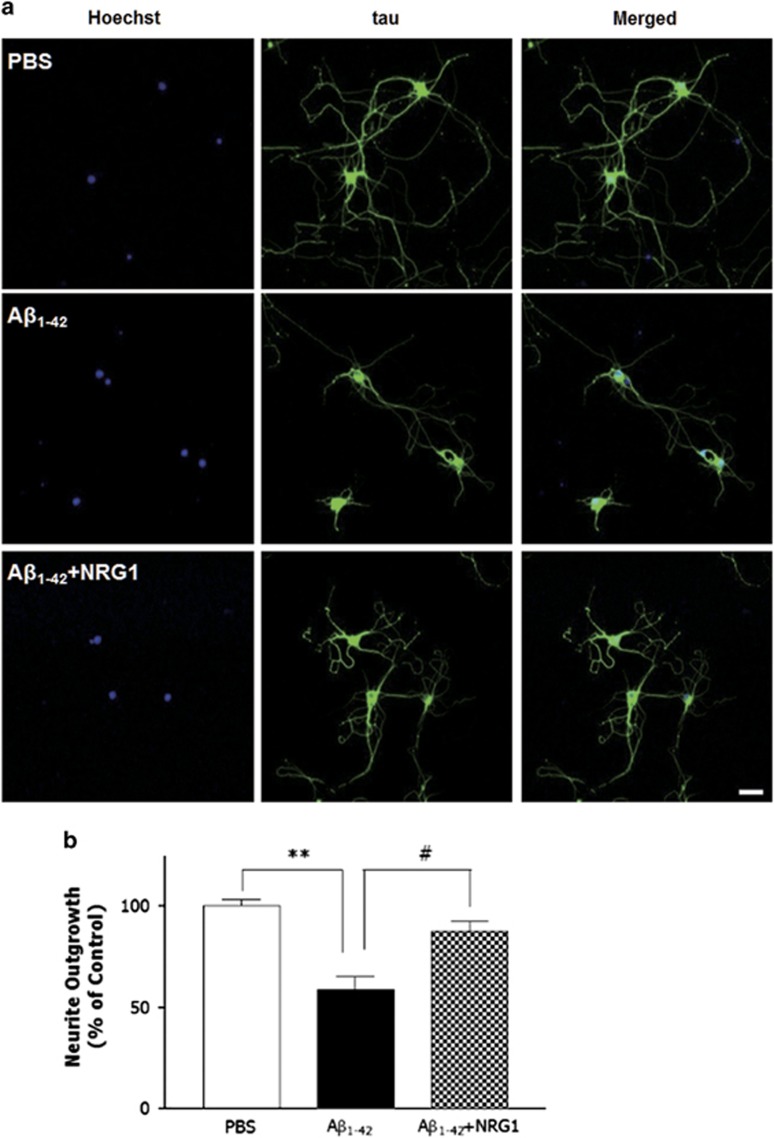
NRG1 has attenuating effect against oligomeric A*β*_1-42_-induced reduction of neurite outgrowth. (**a**) Representative images of neurons stained with anti-tau antibody and visualized with FITC-coupled secondary antibody are shown. Primary hippocampal neurons were treated with 250 nM oligomeric A*β*_1-42_ (at DIV 13) either alone or with PBS or NRG1 (at DIV 14). The neurons were fixed and stained with anti-tau antibody, and visualized with FITC-coupled secondary antibody. Scale bar, 100 *μ*m. (**b**) The values of neurite outgrowth are shown as the percentage of the control group, *n*=6, **P<0.01, ^#^*P*<0.05. Neurite outgrowth in the neurons exposed to oligomeric A*β*_1-42_ (250 nM) for 4 days was reduced significantly by 41.3%. (253.7±23.7 *μ*m, *n*=6, **P<0.01), compared with the PBS-treated controls (363.4±10.0 *μ*m, *n*=6). Co-treatment with 10 nM NRG1 for 3 days restored neurons from oligomeric A*β*_1-42_-induced reduction of neurite outgrowth to 350.7±20 *μ*m (^#^*P*<0.05, *n*=6)

To further determine whether NRG1 affects the decrease in neurite outgrowth induced by oligomeric A*β*_1-42_, we tested the effects of NRG1 in rat primary hippocampal neuron cultures under the same treatment conditions used to evaluate dendritic spine density. As shown in Figures 3a and b, neurite outgrowth in the neurons exposed to oligomeric A*β*_1-42_ (250 nM) for 4 days was reduced significantly (253.7±23.7 *μ*m, *n*=6, **P<0.01) compared with the PBS-treated controls (363.4±10.0 *μ*m, *n*=6) by 41.3%. The co-treatment with 10 nM NRG1 for 3 days restored neurons from oligomeric A*β*_1-42_-induced reduction of neurite outgrowth to 350.7±20 *μ*m (^#^*P*<0.05, *n*=6; Figure 3b). These results indicate that NRG1 has a significant attenuating effect against oligomeric A*β*_1-42_-induced reduction of neurite outgrowth.

### NRG1 rescues the impairment in neural differentiation induced by oligomeric A*β*_1-42_ in fetal neural stem cells

Oligomeric A*β*_1-42_ has been reported to reduce neural differentiation in fetal neural stem cells.^32^ Here, we examined whether NRG1 affected the neural differentiation impairments induced in fetal neural stem cells with 500 nM oligomeric A*β*_1-42_ treatment. Neural stem cells of passage 3 were differentiated in differentiation medium for 3 days. We counted the number of neurons stained with anti-microtubule associated protein-2 (MAP2) or anti-glial fibrillary acidic protein (GFAP) antibodies that were counterstained with the nuclear marker, 4', 6-diamidino-2-phenylindole (DAPI). MAP2 stains processes as well as cell bodies.

**Figure 4:**
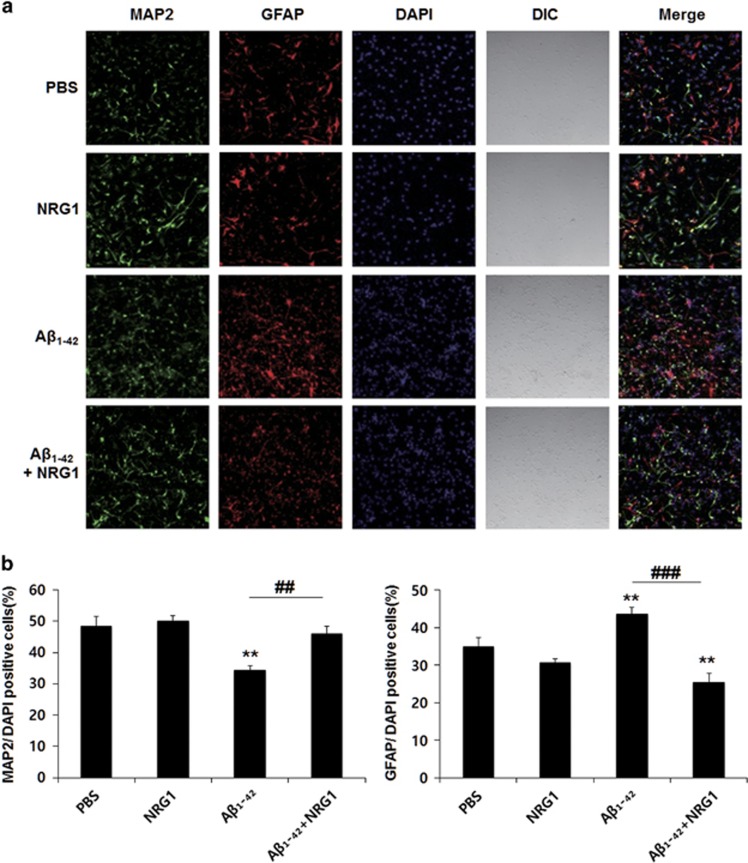
NRG1 attenuates the impairment in neural differentiation induced by oligomeric A*β*_1-42_ in primary fetal neuronal stem cells. (**a**) Representative immunofluorescence image of cultured fetal neuronal stem cells 5 days after treatment with PBS or oligomeric A*β*_1-42_ (500 nM) and either PBS or 10 nM NRG1 at day 2 for 3 days. Cells were fixed on day 5, immunostained for the neuronal marker MAP2 (green) and the astrocytic marker GFAP (red) and counterstained with DAPI (blue). Orthogonal analysis was carried out on the stained cells via confocal microscopy. (**b**) Quantitative graph of MAP2- and GFAP-positive cells (expressed as the percentage of DAPI-positive cells) is shown. Treatment with 500 nM oligomeric A*β*_1-42_ for 3 days reduced neural differentiation (34.452±1.271%, *n*=6, **P<0.01), compared with the PBS-treated control (48.502±3.039%, *n*=6) but increased the GFAP-positive cells (43.625±1.688%, *n*=6, **P<0.01), compared with the control (34.868±2.367%, *n*=6). However, co-treatment with 10 nM NRG1 significantly attenuated the decrease in neural differentiation induced by A*β*_1-42_ treatment (45.866±2.515%, *n*=6, ^##^*P*<0.01). In contrast, co-treatment with 10 nM NRG1 significantly reduced the quantity of GFAP-positive cells (25.479±2.318, *n*=6, ^###^*P*<0.001), compared with the 500 nM A*β*_1-42_ plus PBS-treated cells (43.625±1.688, *n*=6). Data represent the mean±S.E.M. from two independent experiments

We found that treatment with 500 nM oligomeric A*β*_1-42_ for 3 days significantly reduced neural differentiation (34.452±1.271% of DAPI-positive cells, *n*=6, **P<0.01), compared with the PBS-treated control (48.502±3.039% of DAPI-positive cells, *n*=6), but it increased the quantity of GFAP-positive cells (43.625±1.688% of DAPI-positive cells, *n*=6, **P<0.01), compared with the control (34.868±2.367%, *n*=6). However, co-treatment with 10 nM NRG1 significantly attenuated the decrease in neural differentiation induced by A*β*_1-42_ treatment (45.866±2.515% of DAPI-positive cells, *n*=6, ^##^*P*<0.01, Figures 4a and b). In contrast, co-treatment with 10 nM NRG1 significantly reduced the quantity of GFAP-positive cells (25.479±2.318% of DAPI-positive cells, ^###^*P*<0.001), compared with the 500 nM A*β*_1-42_ plus PBS-treated cells (43.625±1.688% of DAPI-positive cells, *n*=6).

## Discussion

Growth factor release via proteolytic processing appears to be an important regulator of many signaling events.^33, 34, 35^ NRG1 signaling is critical for nervous system development, aspects of neuronal migration, synapse formation, gliogenesis and neuronal–glial communication.^4, 36, 37^ In mature animals, alterations in NRG1 signaling manifest as aberrant peripheral myelination and as deficits in synaptic plasticity in hippocampal and cortical slices.^38, 39, 40^

Several lines of evidence have demonstrated that NRG1 protects neurons against toxic stimuli under various conditions including ischemia and exposure to organophosphates.^41, 42, 43, 44^ NRG1 prevented neurons from entering apoptosis following focal cerebral ischemia via inhibition of caspase-3 and TNF-*α* expression. It has been shown that the PI3K/Akt pathway has a major role in neuronal survival after an ischemic insult.^43^ Previously, we reported that NRG1 exerts neuroprotective effects against neurotoxicity induced by Swedish APP and APP-CT overexpression and A*β*_1-42_ in neuronal cells via the ErbB4 receptor.^18, 19^ Furthermore, NRG1 attenuated the A*β*_1-42_-induced impairment of LTP in hippocampal slices via ErbB4.^17^

NRG1 polymorphisms have been identified in families with late onset AD with psychoses. A NRG1 single nucleotide polymorphism, rs392499 (glycine to alanine), in the second exon of the NRG1 gene was found in 65 AD families.^45^ In addition, a recent paper reported that eight single nucleotide polymorphisms in the NRG3 gene were significantly associated with the risk of AD.^46^ These studies provide evidence that NRG may be responsible for the cellular alterations that produce the clinical phenotype of AD.

BACE1 cleavage of APP is one of the first steps in the production of A*β*. BACE1-KO mice are reported to share phenotypes with APP KO mice and NRG1 KO mice.^47^ The close relationship between NRG1, BACE1 and APP suggest a potential link between NRG1 and AD.

AD is clinically diagnosed by the progressive loss of memory and learning impairments.^15^ We investigated whether NRG1 influences the cognitive function in an AD model. Tg2576 mouse is a well-characterized animal model of AD that express Swedish mutant APP.^48^ In this study, spatial memory of 13-month-old WT and Tg2576 mice infused with PBS or NRG1 for 4 weeks was tested by Morris water maze. We selected the age of 13 months (later phase and not the early phase of cognitive deficits) to allow a more obvious measurement of the effects of NRG1. As Figure 1c indicates, WT mice treated with PBS appeared not to remember the location of the platform (zone 4) in the probe test that was performed 1 day after four consecutive days of training. The results show that at 13 months of age, the WT mice successfully learn the location of the platform but have trouble in maintaining memory. NRG1 treatment resulted in improvement in both WT-NRG1 and Tg2576-NRG1 groups. Altogether, NRG1 may have a protective effect in not only AD but also in age-related memory decline.

To investigate whether NRG1 directly affected AD progression, we examined A*β* levels in the hippocampus of WT or Tg2576 mice in the PBS- or NRG1-infused groups. In Tg2576 mice, we observed no significant differences in the level of A*β* between the PBS- and NRG1-infused groups (Supplementary Figure 1). This result suggests that the beneficial effects of NRG1 on the behavioral changes may not be caused by direct effects on A*β* generation or degradation.

Several recent studies have examined the effects of NRG1 on synaptic plasticity or transmission in the CNS. NRG1 heterozygous-mutant mice exhibited a decrease in the number of functional NMDA receptors in the forebrain,^49^ while treatment with NRG1 increased the number of spines in cultured hippocampal neurons.^28^ We measured the effects of NRG1 in both *in vitro* and *in vivo* experimental models of AD and investigated the underlying mechanisms of NRG1 function. NRG1 rescued the reduction in dendritic spine density that was detected in both *in vitro* and *in vivo* models of AD. Previously, it was reported that long-term incubation with NRG1increased the dendritic spine density in cortical pyramidal neurons, and this effect was shown to be mediated by RacGEF kalirin, a well-characterized regulator of dendritic spines.^30^ The effects of NRG1 on dendritic spine density in pyramidal cortical neurons are consistent with our results obtained with primary hippocampal neurons. We also found that NRG1 has an attenuating effect against oligomeric A*β*_1-42_-induced reduction of neurite outgrowth in hippocampal neurons. Therefore, we propose that the advantageous effects of NRG1 on the AD-related behavioral changes may result from its effects on synaptic plasticity, as assessed with dendritic spines and synaptic proteins such as PSD95 in Tg2576 mice that are at least 13 months of age.

Neurogenesis is reduced in several neurodegenerative disorders including AD^50^ and it is believed that induction of neurogenesis by targeting endogenous neural stem cells could be a potential therapeutic approach. We showed that NRG1 rescues the impairment in neural differentiation induced by oligomeric A*β*_1-42_ in fetal neural stem cells. Our results provide strong evidence that NRG1 may exert beneficial effects in AD via affecting neurogenesis.

AD is critically associated with age as a risk factor, and the expression of neurotrophic factors is known to decrease with increasing age.^51^ Collectively, our results suggest that NRG1 ameliorates cognitive dysfunction in AD through several mechanisms and that NRG1 could be developed as a therapeutic treatment for this disease. Because the current study was conducted in old mice with advanced AD, further study is needed to clarify the effects of NRG1 in the early stage of the disease.

## Materials and Methods

### Reagents and antibodies

Recombinant human NRG1/Heregulin-*β*2 (rHuNRG1) was purchased from PROSPEC (East Brunswick, NJ, USA). The A*β*_1-42_ was obtained from Sigma Chemical Company (St. Louis, MO, USA). Rabbit anti-GAPDH polyclonal antibody was purchased from Ab FRONTIER (Seoul, Korea). Rabbit anti-GFAP polyclonal antibody was purchased from DAKO (Carpinteria, CA, USA). Mouse anti-MAP2, clone AP 20 monoclonal antibody was purchased from Millipore (Billerica, MA, USA). Mouse anti-PSD95 clone, 7E3-1B8 monoclonal antibody was purchased from Thermo (Rockford, IL USA); mouse anti-tau monoclonal antibody and rabbit anti-A*β* monoclonal antibodies were purchased from Cell Signaling Technology (Danvers, MA, USA). The fluorescent secondary antibodies, Alexa 488-conjugated goat *α*-mouse IgG and Alexa 555-conjugated goat α-rabbit IgG were obtained from Invitrogen (Carlsbad, CA, USA).

### Transgenic mice

All animal procedures were carried out following the National Institutes of Health Guidelines for the Humane Treatment of Animals, with approval from the Institutional Animal Care and Use Committee of Seoul National University (IACUC No. SNU-121012-03). Tg2576 mice were obtained from Taconic Farms (Germantown, NY, USA) and were bred by mating male mice with C57B16/SJL F1 female mice, as recommended by the suppliers and as described by others.^52^ Comparisons were performed between heterozygous transgenic (Tg2576) mice and age-matched transgene-negative littermates (WT).

### Western blot analysis

Protein (50–80 *μ*g) from hippocampal tissues lysed in RIPA buffer was loaded onto 10% Tris-Glycine gels and transferred to NC membranes (Millipore). Membranes were incubated with the appropriate primary antibodies (anti-A*β*, 1: 1000, Cell Signaling; GAPDH, 1: 2 500, Ab FRONTIER). GAPDH was used as the loading control. In turn, the membrane was incubated with anti-rabbit or anti-mouse secondary antibodies conjugated to horse radish peroxidase (1: 1000, Invitrogen), and are shown using an enhanced chemiluminescent substrate (Ab FRONTIER).

### Intracerebroventricular infusion of NRG1 using an osmotic pump

Alzet model 1004 micro-osmotic pumps (DURECT, Cupertino, CA, USA), with a capacity of 100 *μ*l and an injection rate of 0.11 *μ*l/h for 4 weeks, were used in this study. The osmotic pumps were filled with NRG1 peptide (28 ng/kg) or PBS according to the manufacturer's instructions. The pre-filled pumps were then placed in PBS at 37 °C for 24 h. Mice were fitted with a stainless steel cannula, which was supplied with the Alzet brain infusion kit (DURECT), that was inserted into the right lateral ventricle of the brain according to stereotaxic coordinates. The osmotic pumps were subsequently implanted subcutaneously into the back and connected to the fitted cannula. All surgical procedures were carried out under anesthesia via intramuscular injection of Rompun (17 mg/kg) and Zoletil (12.5 mg/kg). All mice were housed individually during the post-surgical observation period.

### Morris water maze task

Mice performed the Morris water maze task 4 weeks after intracerebroventricular infusion of vehicle (PBS) or NRG1 with an osmotic pump. The experimental apparatus, which was a circular water tank (diameter=140 cm, height=45 cm), was filled with opaque water produced by adding dry milk powder to water at room temperature (21–23 °C). Animals were required to find a submerged platform (diameter=12 cm, height=35 cm) in the pool using spatial cues. Three training trials per day were conducted for four consecutive days, in which the initial placement of the mice into the maze varied in each trial for each group. For each training session, the latency to escape to the hidden platform was recorded. Forth-eight hours after the final trial session, a single probe trial was conducted. The escape platform was removed and each mouse was allowed to swim for 60 s in the maze. The behavioral tests were performed in a blinded manner.

### Tissue preparation

Mice were anesthetized via intramuscular injection of Rompun (17.5 mg/kg) and Zoletil (12.5 mg/kg) and killed after behavioral testing. The mice were immediately perfused intracardially with PBS containing heparin. After perfusion, each brain was extracted and fixed in 4% paraformaldehyde solution for 24 h at 4 °C and incubated in 30% sucrose solution for 48 h at 4 °C for immunohistochemical analysis. Sequential 25 *μ*m coronal sections were generated using a cryostat (Cryotome, Thermo Electron Cooperation, Waltham, MA, USA) and stored at 4 °C.

### Immunohistochemistry

Brain sections were incubated in 0.3% Triton-X 100 for 10 min for permeabilization and blocked with 10% normal goat serum. The sections were incubated with primary antibodies overnight, followed by incubation with appropriate fluorescence-associated secondary antibodies and 1 *μ*M DAPI for 1 h at room temperature. After three washes in permeabilization buffer and a wash in PBS, sections were mounted on microscope slides in mounting medium (DAKO). Confocal microscopic observation was performed using LSM 510 (Carl Zeiss, Jena, Germany). The experimenter was blinded to the groups of each sample.

### Golgi-Cox staining

Golgi-Cox staining was performed using the FD Rapid GolgiStain Kit (FD Neuro Technologies, Columbia, MD, USA), as previously described.^53^ Briefly, animals were anesthetized, and the brains were taken from the skulls and rinsed in double distilled water. The brains were immersed in impregnation solution, and stored at room temperature for 2 weeks in the dark. The impregnation solution was replaced the following day, and the brains were transferred to Solution C. Solution C was replaced the following day and the brains were stored at 4 °C for 3 days in the dark. Brain sections (120 *μ*m thickness) were generated using a Cryotome (Thermo Electron Cooperation) with the chamber temperature set at −22 °C. Each section was mounted on saline-coated microscope slides using Solution C. After the absorption of excess solution, the sections were allowed to dry naturally at room temperature. The dried sections were processed according to the manufacturer's instructions. Briefly, dendrites within the CA1 subregion of the hippocampus were imaged using a × 100 objective using an Olympus BX-51 microscope (Wetzlar, Germany) with a Leica DFC 280 digital camera (Tokyo, Japan). Dendritic spines were detected along CA1 secondary dendrites starting from their point of origin on the primary dendrite and the counting was performed by an experimenter blinded to the group of each sample.

### Primary hippocampal neuron culture

The hippocampus was removed from Sprague–Dawley rat embryos (embryonic day 18). Cells were dissociated with trypsin (0.25%) and then seeded on coverslips which were coated with poly-L-lysine. Cells were transfected with 3 *μ*g of IRES-mGFP vector using CalPhos Mammalian Transfection Kit (Clontech Laboratories, Mountain View, CA, USA). The neurons were cultured in Neurobasal medium (Gibco, Grand Island, NY, USA) supplemented with B27 (Gibco), 2 mM GlutaMAX-I supplement (Gibco) and 100 *μ*g/ml penicillin/streptomycin (Gibco) at 37 °C in a humidified environment with 5% CO_2_.

### Preparation of oligomeric A*β*

We used synthetic A*β*_1-42_ peptides with >95% purity based on RP-HPLC chromatography (American Peptide Company, Sunnyvale, CA, USA). Briefly, the A*β*_1-42_ was dissolved to 1 mM in 100% hexafluoroisopropanol (Sigma Chemical Company). Next, the hexafluoroisopropanol was removed under vacuum, and the peptide was stored at −20 °C. Then, for oligomeric conditions, F-12 (without phenol red) culture medium was added to dissolve the peptide to a final concentration of 100 *μ*M, and the peptide was incubated at 4 °C for 24 h.

### Dendritic spine density analysis

Primary hippocampal neuron cultures (DIV 12) were transfected with IRES-mGFP vector. The number of dendritic spines was evaluated at DIV 17. Fluorescent images were obtained with an LSM 510 confocal microscope (Carl Zeiss) by reusing the same settings for all samples. Spine density was measured from 20- to 40-*μ*m segments of secondary dendrites extending from at least 40–80 *μ*m away from the cell body (soma). All of the analyses were performed in a blinded manner.

### Quantification of neurite outgrowth

For assessing neurite outgrowth, the cultures were fixed with 4% paraformaldehyde in PBS (pH 7.4) for 20 min at room temperature. Fixed cells were then incubated in blocking buffer with 10% normal horse serum and PBS for 1 h and rinsed three times with PBS. Neurons were labeled by indirect immunofluorescence using a primary antibody to tau and an Alexa Fluor 488-conjugated secondary antibody. After immunostaining, confocal microscopy was performed using a Zeiss LSM 510 confocal microscope. Neurite outgrowth was quantified using ZEN 2012 software (Carl Zeiss). All of the procedures were performed in a blinded manner.

### Fetal neural stem cell culture

Fetal neural stem cell culture was carried out as previously described.^54^ Neurospheres derived from fetal neural stem cells were generated as previously described.^55^ For immunocytochemistry analysis, we dissociated the neurospheres and subcultured the fetal neural stem cells (5 QUOTE cells/well) in 24-well plates that contained 12 mm glass coverslips (Marienfeld, Lauda-Konigshofen, Germany) pre-coated with laminin (Roche, Mannheim, Germany) at a concentration of 20 *μ*g/ml in MEM with 1 M HEPES and penicillin/streptomycin. The cells were incubated in the differentiation medium without growth factors (epidermal growth factor and bFGF) for 5 days and were assayed via immunocytochemistry.

### Immunocytochemistry

Cells were fixed with 4% paraformaldehyde for 30 min at room temperature. Then, the cells were washed with PBS, followed by permeabilization with PBS containing 0.05% Tween 20. Next, the cells were blocked with blocking buffer (3% normal goat serum, 2% BSA and 3% Triton-X 100 in PBS) for 1 h at room temperature. After washing, the cells were incubated in PBS containing appropriate primary antibodies for 2 h at room temperature. After the incubation, the cells were washed with PBS. Then, the cells were incubated in PBS containing Alexa Flour 488- or 546-conjugated secondary antibodies, and 1 *μ*M DAPI for 1 h at room temperature. The fluorescence signals were visualized by a confocal microscope (LSM 510, Carl Zeiss).

### Statistical analysis

Quantitative results are described as the means±standard error of the mean (S.E.M.). Student's *t*-test and a one-way ANOVA using Fisher's LSD *post hoc* test were used to analyze statistical significance (IBM SPSS Statistics 20, Chicago, IL, USA). The results were considered statistically significant if *P*<0.05.

## Abbreviations

AD: Alzheimer's disease
A*β*: amyloid beta peptide
APP: amyloid precursor protein
BACE1: *β*-site-APP cleaving enzyme 1
DAPI: 4', 6-diamidino-2-phenylindole
EGF: epidermal growth factor
GAPDH: glyceraldehyde 3-phosphate dehydrogenase
GFAP: glial fibrillary acidic protein
KO: knockout
LTP: long-term potentiation
MAP2: microtubule-associated protein-2
NRG: neuregulin
PBS: phosphate-buffered saline
PSD95: postsynaptic density 95
S.E.M.: means±standard error of the mean
WT: wild-type
