# Supplementary figures and images for: Neuregulin-1 attenuates cognitive function impairments in a transgenic mouse model of Alzheimer's disease
Source: Cell Death Dis. 2016 Feb 25;7(2):e2117–. doi: 10.1038/cddis.2016.30 (PMC4849157; doi:10.1038/cddis.2016.30)

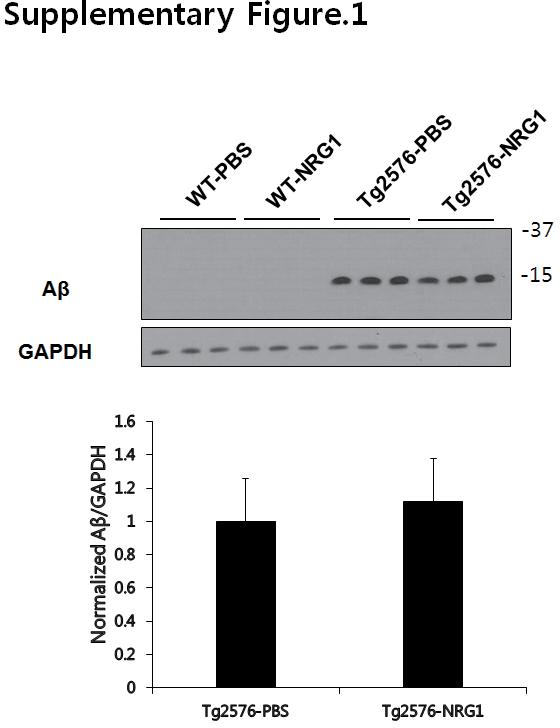

Supplement: Supplementary Figure 1 [file cddis201630x1.tif]
