# Supplementary material for: Neuregulin-1 attenuates cognitive function impairments in a transgenic mouse model of Alzheimer's disease
Source: Cell Death Dis. 2016 Feb 25;7(2):e2117–. doi: 10.1038/cddis.2016.30 (PMC4849157; doi:10.1038/cddis.2016.30)
Supplement: Supplementary Figure Legend [file cddis201630x2.docx]

**Supplementary Figure 1.**

**NRG1 does not affect Aβ level in Tg2576 mice**

Aβ levels were measured by Western blotting in the hippocampi of WT and Tg 2576 mice infused with PBS or NRG1. Aβ levels in the hippocampus of the Tg2576 mice did not differ significantly compared with levels in PBS-infused Tg 2576 mice. Data are presented as the mean ± SEM.
